# Effect of Dietary Palm Kernel Oil on the Quality, Fatty Acid Profile, and Sensorial Attributes of Young Bull Meat

**DOI:** 10.3390/foods11040609

**Published:** 2022-02-21

**Authors:** Neiri J. A. dos Santos, Leilson R. Bezerra, Daniela P. V. Castro, Polyana D. R. Marcelino, Gercino F. Virgínio Júnior, Jarbas M. da Silva Júnior, Elzânia S. Pereira, Ederson A. de Andrade, Thadeu M. Silva, Analívia M. Barbosa, Ronaldo L. Oliveira

**Affiliations:** 1 Department of Animal Science, Federal University of Bahia, Salvador 40170110, Bahia, Brazil; neirijean@hotmail.com (N.J.A.d.S.); danipionorio_zootecnia@hotmail.com (D.P.V.C.); polyana_deyse@hotmail.com (P.D.R.M.); gercino.ferreiravj@yahoo.com.br (G.F.V.J.); miguelreges@gmail.com (J.M.d.S.J.); edersonzootec@gmail.com (E.A.d.A.); thadeumsilva@gmail.com (T.M.S.); analiviabarbosa@gmail.com (A.M.B.); 2 Department of Animal Science, Federal University of Campina Grande, Patos 58708110, Paraiba, Brazil; leilson@ufpi.edu.br; 3 Department of Animal Science, Federal University of Ceará, Fortaleza 60021970, Ceará, Brazil; elzania@hotmail.com

**Keywords:** animal nutrition, lauric acid, lipids, ruminants, shear force

## Abstract

Lipid supplementation through vegetable oils in diets for ruminants can be a nutritional strategy to increase energy density, manipulate ruminal fermentation and change the physicochemical composition and sensorial properties of meat. This study evaluated the optimal dietary inclusion of palm kernel oil (PKO) for Nellore bulls on meat quality. The diets consisted of 0.0, 11.5, 23.0, and 34.6 g/kg dry matter (DM) PKO levels. PKO inclusion did not influence the centesimal composition, pH, color indices, water holding capacity, cooking loss, or shear force of the beef. There were linear increases in the concentrations of lauric acid (C12:0) and myristic acid (C14:0) in the bull’s meat. However, palmitic acid (C16:0), oleic acid (C18:0), vaccenic acid (*t*-11–C18:1) and conjugated linoleic acid (CLA), ∑*n −* 6, ∑*n −* 3, ∑*n −* 6/∑*n* 3, the hypocholesterolemic: hypercholesterolemic ratio of the fatty acid content, and the thrombogenicity index were not affected. There were linear reductions in the oleic acid meat concentration (*c*-9–C18:1) and elongated enzymatic activity when PKO was added to the bull diet. The atherogenicity index increased linearly due to PKO inclusion in the bull diet. No effect of the inclusion of PKO on meat flavor, perception of tenderness, juiciness, or global acceptance from the sensorial evaluation was recorded. The inclusion of PKO up to 34.6 g/kg DM can be recommended to supplement young bulls with no effects on meat composition and quality characteristics.

## 1. Introduction

The use of lipid supplementation through vegetable oils in diets for ruminants is justified by its high energy density [1]. Fatty acids can also be used to manipulate ruminal fermentation by inhibiting the activity of some microorganisms; for example, palm kernel oil exerts an antimicrobial effect [2], promoting changes in the rumen microbiome, which impacts digestion and absorption of nutrients [3]. This change in the profile of fatty acids deposited in meat provides health benefits to humans by promoting the formation of essential fatty acids, including conjugated linoleic acid (CLA), for its nutraceutical properties, such as anticarcinogenic, antiadipogenic, and anti-inflammatory effects [4].

Palm kernel oil (PKO) is extracted from the oil palm fruit (*Elaeis guineenses*), a plant of African origin. In Brazil, oil palm cultivation is favored by the tropical climate present in some regions of the country, such as the North and Northeast regions, in addition to favorable soil conditions [5]. The oil extracted from the pulp of the palm kernel has medium-chain fatty acids (MCFAs) and saturated fatty acids (SFAs), with the highest concentrations of lauric acid (46.6%) and myristic acid (16.0%). Lauric acid destabilizes the cell membrane, disrupting nutrient transport and energy metabolism, resulting in the cell death of microorganisms, such as cellulolytic bacteria and ciliated protozoa [2].

Reducing the population of microorganisms in the rumen environment can interrupt some specific steps of biohydrogenation, promoting a greater passage of unsaturated fatty acids (UFAs) to the small intestine of the animal, which can be absorbed and deposited in muscles [6], changing the meat FA composition. Thus, supplementation with oil in ruminant diets, depending on the amount of lipid, the FA profile of the supplemented source, and the duration of feeding [7,8], can alter the composition of the meat, its nutritional, and physical-chemical characteristics, directly influencing its quality [9]. Therefore, evaluating such characteristics when using oils with an MCFA and SFA profile is important to ensure the product quality was supplied to the consumer market.

Therefore, our study aimed to evaluate the inclusion of PKO in the bull’s diet modifies ruminal biohydrogenation and the FA with an increase in CLA concentrations and sensory acceptance of meat.

## 2. Materials and Methods

### 2.1. Ethics Committee

The experiment was conducted following the animal use and welfare protocols of the Ethics Committee on the Use of Animals (CEUA) of the School of Veterinary Medicine and Animal Science of the Federal University of Bahia, Protocol 01/2015.

### 2.2. Animals, Treatments, and Experimental Design

Thirty-two Nellore bulls (413 ± 29.0 kg) of twenty-four months of age were used. The bulls were individually distributed in partially covered stalls (2.0 × 4.0 m^2^) with concrete floors, feeders, and drinking troughs. The trial lasted 105 days, and the first 15 days were dedicated to adapting the animals to the facilities, management, and diets.

The experimental design was completely randomized (four levels of palm kernel oil and eight replications), and 0.0, 11.5, 23.0, and 34.6 g/kg palm kernel oil (PKO) inclusion based on dry matter (DM) of the diet constituted the treatments.

### 2.3. Diets and Chemical Composition

The diets were isoproteic, isoenergetic, and formulated following the recommendations of the Seventh Revised Edition of the National Research Council [10] and contained 124 g CP/kg DM to meet the nutritional requirements of the bulls for an estimated average daily gain of 1500 g/d. The diets were offered twice a day (8 h and 16 h) as a total mixed ration (TMR) with forage and concentrate ratio (400:600 g/kg DM). Water was provided *ad libitum*. Samples of ingredients and diets were collected and analyzed (Table 1 and Table 2).

### 2.4. Chemical Analysis and Calculations

All collected samples were dried in a forced ventilation oven at 55 °C for 72 h. Then, all samples were ground in Willey mills (Tecnal, Piracicaba, São Paulo, Brazil) on a 1 mm sieve, stored, and identified in plastic pots. All samples were analyzed in duplicate and assuming a coefficient of variation of less than 5% for all analyses.

The analyses were performed following the Association of Analytical Communities [11], and the contents were determined for DM (method 967.03), crude protein (CP—method 981.10), crude ash (MM—method 942.05), and ether extract (EE—method 920.29). The determinations of neutral detergent fiber (NDF) and acid detergent fiber (ADF) followed the methodology by Van Soest et al. [12]. The neutral detergent fiber content was incinerated at 600 °C for 4 h to correct for ash and then submitted to protein analysis for neutral detergent insoluble protein (NDIP) subtraction and protein correction. The contents of NDIP and acid detergent insoluble protein (ADIP) were obtained following Licitra et al. [13]. The content of acid detergent lignin was performed following method 973.18 [11]. Nonfibrous carbohydrates (NFC) were calculated following the equation given by Hall [14]:

NFC = 100 − [(%CP − %CP from urea + %urea) + %NDF_ap_ + %EE + %ash]. (1)

### 2.5. Slaughter and Meat Sampling

The performance data of the animals during the preslaughter period are described in Table 2. At the end of the 105-day trial, the animals were transferred to a slaughterhouse after solid fasting for 16 h. Slaughter was carried out following the guidelines of the Ministry of Agriculture, Livestock, and Supply (Normative n° 03/00, [15]) for the Federal Inspection on Service (FIS) for humane slaughter. The animals were stunned with a pneumatic pistol, followed by bleeding through the jugular veins and carotid arteries, and then skinned and eviscerated. The head and hooves were also removed. Subsequently, the carcasses were half sectioned and placed in a cold chamber (4 °C) for 24 h. After weighing, all left and right *longissimus thoracis* (LT) muscles were dissected, packaged, identified, and stored in a freezer (−20 °C) for further analysis.

### 2.6. Proximate Composition and Physicochemical Characteristics of Meat

The meat pH was measured at 20 °C, using a digital skewer-type probe (Digimed, 300 M, São Paulo, Brazil), before (≈30 min postmortem; 0 h), and 24 h after slaughter, after cooling of the carcass.

The color analysis was measured using a Minolta CR-400 (Konica Minolta, Tokyo, Japan) colorimeter (8 mm aperture; 10° observer) in a new cross-section of muscle cut from the LT sensory sample and allowed to bloom at temperatures between 6–7 °C for 40 min [16]. The colorimeter light source was a pulsed xenon lamp. The aperture port had a glass cover, and the samples were measured using illuminant D65.

Meat color was evaluated after myoglobin oxygenation by exposing the meat cut to the atmosphere for 30 min [17]. The reading was carried out in a Minolta CR-10 colorimeter (Konica^®^ Minolta, Osaka, Japan) using the CIE L*a*b* system to determine the luminosity index (L*), red index (a*) and yellow index (b*). The variables L*, a*, and b* were measured at three different points on the meat surface, and the average was subsequently calculated [18]. The saturation index (chroma) was determined by the red and yellow indices, following the formula [19]:

Chroma = (a*2 + b*2)^0.5^. (2)

The water holding capacity (WHC) of the meat was measured by cutting two-gram cubes and placing them transversely (to the fiber direction) on circular filter paper between two acrylic plates (12 × 12 × 1 cm) for five minutes, receiving a load of 10 kg [20] (Hamm, 1986). Then, the samples were weighed before and after exposure to load, and the WHC values were obtained and expressed as percentages.

Cooking losses (CLs) were determined following the American Meat Science Association [21], evaluating two 2.5-cm-thick samples free from subcutaneous fat. The samples were grilled (George Foreman Jumbo Grill GBZ6BW, Rio de Janeiro, Rio de Janeiro, Brazil) with a stainless-steel thermocouple (Gulterm 700; Gulton Brazil) inserted in the geometric center of each sample until it reached 71 °C. Subsequently, the steaks were removed, exposed to room temperature until the internal temperature stabilized, and weighed again. Finally, the cooking losses were calculated by the difference in weight of the sample before and after cooking, with the value expressed as a percentage. To determine the shear force, the samples were fragmented into three subsamples (1.0 cm in diameter and 2.0 cm in length). The analysis was performed using a texture analyzer (TX-TX2; Mecmesin, NV, USA) with a load of five kgf and a cutting speed of 20 cm/min, using a Warner-Bratzler shear blade, following the methodology by Shackelford et al. [22].

The moisture content, protein, mineral matter, lipids, and collagen were determined by samples of approximately 100 g of fat-free meat ground in a multiprocessor to obtain a homogeneous mass, and then the centesimal composition was determined by proximal infrared analysis [11] using a FoodScan™ (FOSS, Hillerod, Denmark).

### 2.7. Fatty Acid Profiles of Experimental Diets and Meat

Fatty acids were extracted according to Hartman and Lago [23], using a transmethylated simultaneously with hexane and a mixture of methanol/acetyl chloride (20:1 *v*/*v*). The transmethylated samples were analyzed in a gas chromatograph (GC Finnigan Focus, Varian, CA, USA) with a flame ionization detector and a capillary column (SLB-ILL111, 100 m long, 0.25 μm internal diameter, and a 0.20-μm-thick film, Sigma–Aldrich, St. Louis, MO, USA). Hydrogen was used as the carrier gas at a 2.0 mL/min flow rate. The initial oven temperature program was programmed at 70 °C, remaining at this temperature for 4 min, then progressively elevated (13 °C/min) to 150 °C, and maintained for 39 min. Again, the temperature was progressively elevated (10 °C/min) to 215 °C, and maintained for 10 min, for a total of 65 min per sample analyzed. During the analysis, the vaporizer temperature was kept at 250 °C, and the detector was kept at 300 °C.

Samples of the esterified extract and standard were injected into the chromatograph, along with an internal standard (Crotonic Acid, Sigma–Aldrich, St. Louis, MO, USA). FAMEs were identified by comparing the retention times of FAMEs with the respective standards (FAME Mix, C4-C24, Sigma–Aldrich, St. Louis, MO, USA). For the quantification of FAMEs, a response factor was generated for each FA based on the sample, the standard, and the response factor of each FA. The results were quantified by normalizing the areas of the methyl esters, with results expressed as g/100 g FAME. From the identified fatty acid profile, the sums of saturated fatty acids (SFA), monounsaturated fatty acids (MUFA), polyunsaturated fatty acids (PUFA), omega 6 (*n* − 6), omega 3 (*n* − 3), and FA were calculated and defined as the PUFA: SFA and Ʃ*n*–6/Ʃ*n*–3 ratios.

To assess the nutritional quality of the lipidic profile, the atherogenicity (AI) and thrombogenicity (TI) indices were calculated following the Ulbricht and Southgate [24]. The ratio of hypocholesterolemic and hypercholesterolemic FA (h:H) was determined as recommended by Arruda et al. [25]. The activity indices of elongase and Δ9–desaturase enzymes in FA with 16 and 18 carbons were determined following Smet et al. [26].

### 2.8. Sensory Characteristics of Meat

Beef samples from the LL muscle on each side of the carcass were prepared and stored at −20 °C until further analysis. Beef preparation resulted in four replicate steaks per side of the carcass (*n* = 8; *n* steaks = 256), totaling 880 grill cubes. Consumer appeal was assessed using a panel consisting of 110 untrained tasters [21], with 55 women and 55 men (aged 18 and 54 years).

The sensory characteristics of the meat were evaluated by the effective method on a nine-point structured hedonic scale (1, I disliked it a lot to 9, I liked it a lot). The meat samples were placed on an electric grill (George Foreman Jumbo Grill GBZ6BW, Rio de Janeiro, Rio de Janeiro, Brazil) and cooked as described before. After cooking, the steaks were cut into 2-cm^2^ cubes, grouped by treatments, coded with three numerical digits, and transferred to a water bath (75 °C) covered with aluminum foil to keep warm and prevent the loss of volatile aromatic compounds until sensorial analyses were carried out.

The tests were conducted between 9 h and 12 h, with 10 participants per session (11 sessions) with an approximate duration of 15 min. The sessions consisted of ten tasting rounds of booth tastings at pre-established times. Each taster received eight meat samples (4 treatments in duplicate), randomly distributed and coded. The tasters were allocated in individual cabins and received water and cracker-type cookies to consume between tastings to remove the aftertaste. Consumer panels evaluated the following parameters: flavor, tenderness, juiciness, and overall acceptance.

### 2.9. Statistical Analysis

A completely randomized design with four treatments (PKO inclusion at levels 0, 11.5, 23.0, and 34.6 g/kg DM) and eight replicates was adopted. Each bull was considered an experimental unit. The data were analyzed by SAS procedures [27]. The experiment was conducted in a completely randomized design. The following model was used:

*Yij* = *μ + si + eij*, (3)

where *Yij* = observed value; *μ* = general average; *si* = effect of palm kernel oil level (0; 11.5; 23.0 and 34.6 g/kg DM, respectively); and *eij* = effect of experimental error. Polynomial contrasts were used to determine the linear and quadratic effects of different treatment levels, and initial weight was used in the statistical model as a covariate when significant.

A linear mixed model was used for sensory characteristics to analyze each sample’s tenderness, juiciness, flavor, and overall acceptance to identify the factors influencing the response. The fixed effects in the model included the diet (level of PKO), and the random effects in the model included tasters and sessions. The mean values obtained from the sensorial analysis were compared by Tukey’s test. Significance was considered when the value of *p* < 0.05. The regression models with the best data fit were chosen to according to the lowest RMSE value for each variable.

## 3. Results

There was no quadratic effect for any of the analyzed variables in this study, and the values were not included in the tables.

### 3.1. Composition and Physicochemical Characteristics of Meat

The inclusion of PKO did not influence the percentage of moisture or the crude protein concentrations, total lipids, crude ash, and collagens from the meat of the bulls. Likewise, the inclusion of PKO did not change the mean pH, luminosity, a* (*p* = 0.756), b*, or chroma (C*) color index (Table 3). There was no effect of including PKO in the bulls’ diet on water holding capacity (WHC), cooking loss (CL), or shear force (WBSF).

### 3.2. Profiles of Fatty Acids, Nutraceutical Compounds, and Sensorial Characteristics of Meat

Linear increases (*p* < 0.001) in the concentrations of lauric acid (C12:0) and myristic acid (C14:0) were observed in the meat of bulls fed due to PKO inclusion in the diet (Table 4). However, palmitic acid (C16:0) and oleic acid (C18:0) were not affected. There was a linear reduction (*p* = 0.008) in oleic acid content (*c*-9–C18:1). However, palmitoleic acid (*c*-9–C16:1) and vaccenic acid (*t*-11–C18:1) were not affected.

The inclusion of PKO oil in the bull diet did not influence the contents of linoleic acid (C18:2*n* − 6), linolenic acid (C18:3*n* − 3), conjugated linoleic acid (CLA), or arachidonic acid (C20:4*n* − 6) in the meat of the bulls. Similarly, the inclusion of PKO did not alter the other PUFAs.

There was no effect of including palm kernel oil in the bulls’ diet on the sums of SFA, MUFA, PUFA, and the PUFA/SFA ratio, as well as the Ʃ *n* − 6, Ʃ *n* − 3 the Ʃ*n* − 6/Ʃ*n* − 3 ratio, the h/H ratio, and the thrombogenicity index (TI. However, the atherogenicity index (AI; *p* = 0.047) increased linearly. In contrast, elongase enzymatic activity linearly reduced (*p* = 0.028) with the addition of PKO in the bull diets. The activities of the enzymes Δ9–desaturase C16 and Δ9–desaturase C18 were not affected by the inclusion of PKO.

There was no effect (Table 5) of including PKO in the bulls’ diet on meat flavor (*p* = 0.517), tenderness (*p* = 0.527), juiciness (*p* = 0.460) or overall acceptance (*p* = 0.578).

## 4. Discussion

Palm kernel oil (PKO) is a byproduct extracted from the fruit of palm oil, rich in medium-chain fatty acids (MCFAs) and saturated fatty acids (SFAs), with the highest concentrations of lauric acid and myristic acid [2,5]. This co-product can replace expensive lipid sources for dietary inclusions by providing fatty acids to be evaluated for meat deposition and quality.

### 4.1. Composition and Physicochemical Characteristics of Meat

The inclusion of PKO did not significantly change the meat’s proximate composition and physicochemical characteristics. The moisture content of meat varies according to fat deposition in the tissue [28], and there was a similarity in the concentrations of total lipids in the meat; however, the moisture content was also not affected by the diet.

The final pH of the meat is associated with glycogen stores in the muscle. This content, in turn, depends on preslaughter management, so animals that suffer from preslaughter stress tend to use these glycogen stores. In addition to stress, the amount of muscle glycogen can also be influenced by the diet with a consequent influence on the final pH [29,30]. However, the animals were not subjected to any preslaughter stress, or if they were, it occurred equitably; thus, the inclusion of PKO in the diet may not have affected glycogen storage, with no differences between the treatments. At 24 h after slaughter, the pH reached values between 5.45 and 5.53, a normal characteristic of postmortem development and meat quality [31].

The similarity found in the pH of meats with and without PKO, no differences were found in cooking losses, water holding capacity, and color, with color being considered the main selection attribute by consumers [32]. No significant effect was observed on meat color, and all values were by the acceptability standard of consumers. The L* value corresponds to the brightness of the meat, with values equal to or greater than 34 is considered acceptable by consumers [33].

High pH activates the actions of muscle enzymes that use oxygen, causing less myoglobin oxidation, correlating with an increase in the a* color index. As the pH remained at adequate levels (5.45–5.53), no changes were found in the a* index, which presented an index between 22.2 and 22.9, considered adequate for beef samples [34]. The b* yellow content is related to total lipids in the meat composition. Since the amount of fat was not altered, along with its pigments such as xanthophylls and carotenes, no alterations were found in b*, which presented an index between 5.71 and 6.59, which was within the quality standard [34].

Shear force indicates the tenderness of the final product. In the present study, PKO inclusion promoted a mean shear force of 3.44 kgf/cm^2^ (ranging from 3.31 to 3.59 kgf/cm^2^), presenting the characteristics of meat with sensitive tenderness (3.2 to 3.9 kgf/cm^2^; [35]. The inclusion of PKO in the bulls’ diets also did not influence the parameters of CL and WHC, and the mean values obtained were consistent with the meat quality standards.

### 4.2. Profile of Fatty Acids and Nutraceutical Compounds

The inclusion of PKO in diets for bulls increased the contents of C12:0 and C14:0 FA in the bull’s meat. The increase in PKO levels caused a higher percentage in the diet and availability for the animals, with 1.52, 16.5, 28.5 and 36.0 g/100 g FAME for C12:0 and 0.60, 5.82, 10.7 and 13.0 g/100 g FAME for C14:0 in the respective treatments, reflecting a greater deposition of these FAs in meat. According to Silva et al. [36], the SFA content in meat is directly related to the energy level of the diet, so increasing levels of PKO favored the deposition of C12:0 and C14:0 in meat. Despite the lower activity of the elongase enzyme, the concentrations of palmitic and stearic acids did not change; however, this enzyme added two carbon atoms to palmitic acid, resulting in C18:0 [37], and could result in reduced C18:0 concentrations.

The reduction observed for the concentrations of *c-*9–C18:1 suggests that the diet’s composition probably affects the ruminal biohydrogenation process. The inclusion of PKO in the bull’s diet resulted in lower concentrations of C18:1 cis-9, reflecting the reduction of this FA in the diets, which ranged from 32.4 to 32.2 g/100 g FAME, since Δ9–desaturase C18 was not affected, as this enzyme is essential for the synthesis of monounsaturated fatty acids [38]. This reduction of *c-*9–C18:1 in meat is not beneficial to human health, as this fatty acid is considered antiatherogenic, as it works by reducing LDL levels and concomitantly increasing HDL levels in the blood [39]. In addition to reducing the risk of heart disease, consumption of *c-*9–C18:1 also results in a reduced risk of developing type 2 diabetes [40].

The ∑SFA, ∑MUFA, and ∑PUFA meat concentrations were not affected by the increasing PKO concentrations in the bull diets. PUFAs are considered beneficial to consumer health [41], emphasizing ∑*n* − 3, as their consumption reduces the risk of cardiovascular disease [42]. The ∑*n* − 6: ∑*n* − 3 ratio (2-3:1) observed in this study agrees with the ratio reviewed and described by Zárate et al. [43], in which ∑*n* – 6: ∑*n* − 3 ratio values of 2-4:1 were associated with reduced risks of breast, prostate, colon, and kidney cancer.

In contrast, SFAs, mainly lauric, myristic and palmistic, and myristic acids, which have four times the potential to increase cholesterol, are considered atherogenic, a fact that culminated in the increase in the AI, demonstrating the potential for stimulating platelet aggregation; that is, the lower the AI values, the better for the consumer, therefore, the greater the potential prevention of the onset of coronary heart disease [44]. Despite this increase in the concentration of the AI, the analysis of this parameter does not reflect damage to consumer health, as in this study, the AI ranged from 0.69 to 0.89 below the average of 1.00 reported by Ulbricht and Southgate [24].

The effects of SFAs on human health are still inconclusive, as benefits from reducing the consumption of SFAs are only obtained when MUFAs and/or PUFAs replace them because when replaced by refined carbohydrates, the risk of heart disease remains the same [45].

### 4.3. Sensory Attributes of Meat

The sensorial characteristics of the meat of bulls fed diets containing PKO did not differ according to the evaluation of the tasters on the attributes of flavor, tenderness, and juiciness. It is important to highlight that the moisture and lipid contents in the meat are related to juiciness, tenderness, and flavor [46], as there was no significant effect on these parameters, and they were not reflected in the sensory evaluation. Another parameter that affects meat tenderness is the collagen content [47]; the increase in the perception of tenderness is associated with lower collagen content in the muscle. As collagen content was also not influenced by diet, sensitivity was not significantly affected.

## 5. Conclusions

Palm kernel oil is a potential byproduct for inclusion in diets for young bulls. Considering the meat quality data presented, the inclusion of palm kernel oil up to 34.6 g/kg DM can be used in the diet of bulls without altering the proximal composition or the physicochemical and sensorial characteristics of the meat. Furthermore, the changes observed in the nutritional quality of the meat are not detrimental to the health of the consumer.

## Figures and Tables

**Table 1:** Chemical composition of ingredients used in experimental diets (g/kg DM or as stated).

Item	Tifton 85 Hay	Ground Corn	Soybean Meal	Palm Kernel Oil
Dry matter, (g/kg as fed)	901	904	869	992
Crude ash	68.7	12.9	68.2	-
Organic matter	931	987	932	-
Crude protein	70.5	75.8	485	-
^1^ NDIP (g/kg CP)	138	140	120	-
^2^ ADIP (g/kg CP)	118	41.5	27.3	-
Ether extract	10.0	52.0	12.6	994
^3^ NDF_ap_	713	135	154	-
Acid detergent fiber	245	17.4	70.6	-
Nonfibrous carbohydrates	138	724	294	-
Hemicellulose	468	118	83.4	-
Cellulose	188	10.6	62.3	-
Acid detergent lignin	56.7	6.80	8.30	-

^1^ Neutral detergent-insoluble protein; ^2^ Acid detergent-insoluble protein; ^3^ Neutral detergent fiber corrected for ash and protein.

**Table 2:** The proportion of ingredients, the chemical composition of diets (g/kg DM or as stated), and animal performance of bulls fed diets containing levels of palm kernel oil.

Item	Palm Kernel Oil Levels (g/kg DM)			
	0.0	11.5	23.0	34.6
Ingredients				
Ground corn	544	530.7	517.4	504
Soybean meal	26.0	27.8	29.6	31.4
Palm kernel oil	0.00	11.5	23.0	34.6
Mineral mixture ^1^	15.0	15.0	15.0	15.0
Urea + ammonium sulfate ^2^	15.0	15.0	15.0	15.0
Tifton 85 hay	400	400	400	400
Chemical composition				
Dry matter, (g/kg as fed)	905	906	907	908
Crude ash	66.3	66.2	66.2	66.1
Organic matter	934	934	934	934
Crude protein	124	124	124	124
NDIP ^3^	231	230	228	226
ADIP^4^	7.05	7.00	6.95	6.90
Ether Extract	32.6	43.4	54.1	65.0
NDF_ap_^5^	363	361	359	358
Acid detergent fiber	109	109	109	109
Nonfibrous carbohydrates	441	432	423	414
Hemicellulose	254	252	250	249
Celullose	82.8	82.8	82.7	82.7
Acid detergent lignin	26.6	26.5	26.4	26.4
Fatty acid (g/100 g FAME ^6^)				
C12:0	1.52	16.5	28.5	36.0
C14:0	0.60	5.82	10.7	13.0
C16:0	18.1	15.4	12.6	11.4
C18:0	2.38	2.30	3.28	3.23
C16:1 *cis*-9	0.19	0.13	0.16	0.05
C18:1 *cis*-9	32.4	25.1	24.5	22.2
C18:2 *cis*-9, *cis*-12	40.4	30.5	18.2	11.9
C18:3 *cis*-9, *cis*-12, *cis*-15	0.78	0.69	0.15	0.10
Animal performance				
Initial weight (kg)	432	418	401	404
Final weight (kg)	534	538	494	457
Dry matter intake (kg/day)	10.3	10.1	7.47	5.41
Average daily gain (kg/d)	1.14	1.34	1.03	0.59

^1^ Guaranteed levels (per kg in active elements): calcium (max) 220.00 g; (min) 209.00 g phosphorus 163.00 g; sulfur 12.00 g; magnesium 12.50 g; copper 3500.00 mg; cobalt 310.00 mg; iron 1960.00 mg; iodine 280.00 mg; manganese 3640.00 mg; selenium 32.00 mg; zinc 9.000,00 mg; maximum fluorine 1630.00 mg; ^2^ Mixture of urea and ammonium sulfate in a ratio of 9:1; ^3^ Neutral detergent-insoluble protein; ^4^ Acid detergent-insoluble protein; ^5^ Neutral detergent fiber corrected for ash and protein; ^6^ Fatty acid methyl esters.

**Table 3:** Evaluation of the linear effect of composition and physicochemical characteristics of meat from bulls fed diets containing levels of palm kernel oil.

Variables	Palm Kernel Oil Levels (g/kg DM)				SEM ^1^	*p-*Value ^2^
	0.0	11.5	23.0	34.6		Lin
Centesimal composition						
Moisture, g/kg meat	73.0 ± 1.24	73.2 ± 0.58	72.9 ± 0.62	74.3 ± 0.60	0.29	0.114
Protein, g/kg meat	22.9 ± 1.20	23.2 ± 1.05	22.9 ± 0.50	22.1 ± 0.57	0.31	0.564
Lipid, g/kg meat	2.65 ± 0.47	2.25 ± 0.64	2.45 ± 0.43	2.12 ± 0.45	0.18	0.573
Ash, g/kg meat	1.45 ± 0.48	1.38 ± 0.26	1.82 ± 0.70	1.48 ± 0.62	0.19	0.270
pH	5.51 ± 0.22	5.47 ± 0.18	5.45 ± 0.17	5.46 ± 0.21	0.07	0.661
Color parameters						
L* (lightness)	37.9 ± 3.58	37.7 ± 1.76	38.5 ± 2.48	37.0 ± 2.09	0.90	0.579
a* (redness)	22.3 ± 2.03	22.7 ± 1.19	22.8 ± 0.74	22.2 ± 1.48	0.52	0.756
b* (yellowness)	6.51 ± 1.43	6.57 ± 0.77	6.49 ± 0.76	5.64 ± 1.15	0.38	0.126
C* (saturation)	26.5 ± 2.09	26.7 ± 1.17	26.8 ± 0.89	25.8 ± 1.58	0.53	0.398
WHC ^3^, %	73.0 ± 3.29	73.3 ± 1.74	73.2 ± 3.17	72.6 ± 3.84	1.50	0.804
Cooking loss ^4^	24.4 ± 2.09	24.8 ± 2.77	25.4 ± 2.63	25.6 ± 4.01	1.65	0.572
WBSF ^5^ (*N*)	3.37 ± 2.09	3.29 ± 1.20	3.49 ± 0.88	3.59 ± 1.11	0.40	0.599
Collagen (%)	1.55 ± 0.23	1.55 ± 0.16	1.51 ± 0.23	1.42 ± 0.19	0.07	0.198

^1^ Standard error of the mean; ^2^ Significance at *p* < 0.05, Lin, linear, Quad, quadratic effect; ^3^ Water holding capacity; ^4^ g/kg meat exuded; ^5^ Warner–Bratzler shear force (kgf/cm^2^).

**Table 4:** Evaluation of the linear effect of fatty acid composition (g/100 g FAME) of bull meat fed diets containing levels of palm kernel oil.

Variables	Palm Kernel Oil Levels (g/kg DM)				SEM ^1^	*p-*Value ^2^
	0.0	11.5	23.0	34.6		
Saturated fatty acids (SFA)						
C12:0	0.07 ± 0.01	0.12 ± 0.03	0.25 ± 0.10	0.38 ± 0.06	0.03	<0.001
C14:0	2.93 ± 0.24	3.36 ± 0.58	4.37 ± 0.44	4.99 ± 0.22	0.22	<0.001
C16:0	24.9 ± 1.11	27.3 ± 2.83	25.0 ± 0.68	25.3 ± 0.62	1.12	0.854
C18:0	15.6 ± 1.68	15.7 ± 2.75	14.9 ± 2.73	14.4 ± 1.94	1.03	0.357
Monounsaturated fatty acids (MUFA)						
* c-*9–C16:1	3.41 ± 0.30	3.38 ± 0.42	3.77 ± 0.63	3.90 ± 0.50	0.21	0.074
* t-*11–C18:1	1.12 ± 0.17	1.03 ± 0.17	1.40 ± 0.39	1.37 ± 0.07	0.10	0.155
* c-*9–C18:1	39.2 ± 2.34	34.5 ± 2.32	36.0 ± 2.91	32.2 ± 1.00	1.42	0.008
Polyunsaturated fatty acids (PUFA)						
C18:2*n*–6	3.83 ± 0.99	5.10 ± 1.41	3.80 ± 1.00	4.37 ± 0.76	0.64	0.915
C18:3*n*–3	0.26 ± 0.08	0.28 ± 0.17	0.22 ± 0.05	0.31 ± 0.19	0.05	0.501
CLA ^3^	0.23 ± 0.03	0.22 ± 0.07	0.24 ± 0.04	0.26 ± 0.01	0.02	0.393
C20:3*n* − 6	0.21 ± 0.07	0.30 ± 0.14	0.24 ± 0.07	0.37 ± 0.13	0.04	0.108
C20:4*n* − 6	1.09 ± 0.42	1.14 ± 0.48	1.22 ± 0.42	1.72 ± 0.65	0.28	0.134
C20:5*n* − 3	0.20 ± 0.09	0.33 ± 0.19	0.23 ± 0.05	0.39 ± 0.11	0.06	0.122
C22:5	0.45 ± 0.15	0.74 ± 0.23	0.51 ± 0.13	0.85 ± 0.24	0.11	0.100
C22:6*n* − 6	0.05 ± 0.04	0.08 ± 0.07	0.07 ± 0.03	0.14 ± 0.09	0.03	0.102
Summations and ratios						
∑SFA	45.4 ± 1.43	48.5 ± 4.38	46.9 ± 4.01	47.6 ± 2.21	1.96	0.563
∑MUFA	47.1 ± 2.63	42.4 ± 4.18	45.4 ± 4.65	41.9 ± 1.31	1.68	0.121
∑PUFA	6.37 ± 1.72	8.26 ± 3.37	6.56 ± 1.76	9.12 ± 1.87	1.02	0.170
∑PUFA:∑SFA	0.14 ± 0.04	0.18 ± 0.06	0.14 ± 0.04	0.19 ± 0.05	0.02	0.300
Ʃ*n* − 6	1.32 ± 0.21	1.47 ± 0.37	1.48 ± 0.28	1.83 ± 0.58	0.27	0.255
Ʃ*n* − 3	0.52 ± 0.19	0.70 ± 0.14	0.52 ± 0.12	0.83 ± 0.22	0.12	0.239
∑*n* − 6:∑*n* − 6	2.54 ± 0.20	2.10 ± 0.34	2.84 ± 0.25	2.20 ± 0.40	0.50	0.976
Health indexes						
AI ^4^	0.69 ± 0.08	0.84 ± 0.07	0.83 ± 0.10	0.89 ± 0.12	0.06	0.047
TI ^5^	1.53 ± 0.22	1.79 ± 0.35	1.62 ± 0.30	1.59 ± 0.28	0.15	0.998
h/H ^6^	1.61 ± 0.10	1.42 ± 0.24	1.43 ± 0.15	1.33 ± 0.08	0.09	0.176
Enzymatic activity						
Δ9–desaturase C16	12.1 ± 0.83	11.3 ± 2.33	13.1 ± 2.09	13.3 ± 1.37	0.78	0.132
Δ9–desaturase C18	71.5 ± 2.91	68.6 ± 3.31	70.6 ± 5.91	69.2 ± 3.06	2.15	0.620
Elongase	65.9 ± 1.69	62.2 ± 2.14	63.9 ± 1.10	61.5 ± 1.56	1.09	0.028

^1^ Standard error of the mean; ^2^ Significance at *p* < 0.05, linear effect; ^3^ Conjugated linoleic acid (C18:2 cis-9, trans-11); ^4^ Atherogenicity index; ^5^ Thrombogenicity index; ^6^ Hypocholesterolemic and hypercholesterolemic ratio of the fatty acids content.

**Table 5:** Evaluation of the linear effect of sensorial attributes of meat from bulls fed diets with different levels of inclusion of palm kernel oil in the diet.

Attributes	Palm Kernel Oil Levels (g/kg DM)				SEM ^1^	*p*-Value ^2^
	0.0	11.5	23.0	34.6		
Flavor	6.70 ± 0.94	6.85 ± 0.76	6.16 ± 0.75	6.78 ± 0.91	0.16	0.517
Tenderness	5.97 ± 1.54	6.30 ± 1.31	5.27 ± 0.82	6.55 ± 1.45	0.25	0.527
Juiciness	6.13 ± 1.28	6.34 ± 0.89	5.68 ± 1.06	6.58 ± 1.01	0.21	0.460
Overall acceptance	6.45 ± 1.10	6.60 ± 0.99	5.95 ± 0.69	6.83 ± 1.10	0.20	0.578

^1^ Standard error of the mean; ^2^ Significance at *p* < 0.05, linear effect.

## Data Availability

The data that support the findings of this study are available from the corresponding author upon reasonable request.
